# Population Genetics of *Streptococcus dysgalactiae* Subspecies *equisimilis* Reveals Widely Dispersed Clones and Extensive Recombination

**DOI:** 10.1371/journal.pone.0011741

**Published:** 2010-07-23

**Authors:** David J. McMillan, Debra E. Bessen, Marcos Pinho, Candace Ford, Gerod S. Hall, José Melo-Cristino, Mário Ramirez

**Affiliations:** 1 Bacterial Pathogenesis Laboratory, The Queensland Institute of Medical Research and Griffith Medical Research College, Herston, Queensland, Australia; 2 Department of Microbiology and Immunology, New York Medical College, Valhalla, New York, United States of America; 3 Instituto de Microbiologia, Instituto de Medicina Molecular, Faculdade de Medicina, Universidade de Lisboa, Lisboa, Portugal; University of Hyderabad, India

## Abstract

**Background:**

*Streptococcus dysgalactiae* subspecies *equisimilis* (SDSE) is an emerging global pathogen that can colonize and infect humans. Although most SDSE isolates possess the Lancefield group G carbohydrate, a significant minority have the group C carbohydrate. Isolates are further sub-typed on the basis of differences within the *emm* gene. To gain a better understanding of their molecular epidemiology and evolutionary relationships, multilocus sequence typing (MLST) analysis was performed on SDSE isolates collected from Australia, Europe and North America.

**Methodology/Principal Findings:**

The 178 SDSE isolates, representing 37 *emm* types, segregate into 80 distinct sequence types (STs) that form 17 clonal complexes (CCs). Eight STs recovered from all three continents account for >50% of the isolates. Thus, a small number of STs are highly prevalent and have a wide geographic distribution. Both ST and CC strongly correlate with group carbohydrate. In contrast, eleven STs were associated with >1 *emm* type, suggestive of recombinational replacements involving the *emm* gene; furthermore, 35% of the *emm* types are associated with genetically distant STs. Data also reveal a history of extensive inter- and intra-species recombination involving the housekeeping genes used for MLST. Sequence analysis of single locus variants identified through goeBURST indicates that genetic change mediated by recombination occurred ∼4.4 times more frequently than by point mutation.

**Conclusions/Significance:**

A few genetic lineages with an intercontinental distribution dominate among SDSE causing infections in humans. The distinction between group C and G isolates reflects recent evolution, and no long-term genetic isolation between them was found. Lateral gene transfer and recombination involving housekeeping genes and the *emm* gene are important mechanisms driving genetic variability in the SDSE population.

## Introduction

Most streptococci displaying β-hemolysis fall within the pyogenic branch of the 16S rRNA-based taxonomy, and are pathogens or commensals of mammalian hosts [1], [2]. Two species within the pyogenic branch - *Streptococcus dysgalactiae* subspecies *equisimilis* (SDSE) and *Streptococcus pyogenes* (group A streptococcus, GAS) - colonize and/or infect the respiratory tract and skin of the human host [2]. Whereas GAS is an important human pathogen, SDSE is largely considered to be a commensal organism. However, numerous studies report that SDSE can cause disease among otherwise healthy individuals [2], [3], [4], [5]. The disease spectrum of SDSE infection is similar to that of GAS, and includes pharyngitis, post-streptococcal glomerulonephritis, cellulitis, necrotizing fasciitis, septicemia, and streptococcal toxic shock syndrome [6], [7], [8], [9]. Furthermore, in some geographic regions where streptococcal diseases are endemic, surveillance studies report higher rates of throat colonization by SDSE than by GAS [10], [11].

The surface-exposed Lancefield group carbohydrate is an important cell wall antigen that aids in distinguishing between several of the β-hemolytic streptococcal species. *S. pyogenes* almost exclusively expresses the group A carbohydrate [1]. Although the vast majority of SDSE isolates have group G carbohydrate, and are often referred to as group G streptococci, a significant minority of SDSE isolates have group C carbohydrate; very rarely do SDSE harbor the group A or L carbohydrate [1], [12]. Among the GAS and SDSE populations, differences in the sequences of individual *emm* genes are widely used for intra-species strain typing. At present, >200 GAS and ∼50 SDSE *emm* types are recognized (http://www.cdc.gov/ncidod/biotech/strep/strepblast.htm). Although one report finds SDSE isolates expressing *emm* types stg2078 or stg10 to have enhanced invasive disease potential [5], most studies have failed to uncover disease associations among SDSE *emm* types [13], [14], [15]. In contrast, associations between *emm* type and specific diseases are well established for GAS [16], [17], [18], [19].

Comparative genome hybridization studies using a microarray containing probes corresponding to genes encoding virulence factors and putative surface proteins failed to reveal clear cut associations between *emm* type and gene content among SDSE [13]. Multilocus sequence typing (MLST) is a nucleotide sequence-based method that uses core housekeeping genes to characterize genetic relationships between isolates of the same species. MLST has been used extensively to study the β-hemolytic GAS [20], [21], [22] and *S. agalactiae* [23], [24] populations, and was recently used to investigate genetic relationships amongst 61 geographically restricted SDSE isolates [25]. In the present study, an intercontinental collection of SDSE isolates is characterized by MLST and *emm* typing, and the geographic distribution of the identified clones and their genetic relationships are defined.

## Results

### Molecular typing

MLST was used to characterize 117 SDSE isolates collected from three continents (Table S1). Most of the isolates selected for MLST were derived from large independent collections and were chosen, in part, based on prior knowledge of their *emm* type and geographic site of isolation, with the goal being the compilation of a genetically diverse data set.

The isolates represent 24 of the ∼50 known SDSE *emm* types, and include strains bearing the group G or group C carbohydrate. A summary of the epidemiological features of the isolates is provided in Table 1.

**Table 1:** Characteristics of SDSE isolates included in this study.

Collection site	No. of isolates	No. of different *emm* types	Diversity index, *D* a (95% C.I.)^b^	No. of different STs	Diversity index, *D* (95% C.I.)	STs unique to collection site	No. of isolates with group G carbohydrate	No. of isolates with group C carbohydrate	No. of invasive isolates	No. of non-invasive isolates
**Australia**	55	17	0.926 (0.901–0.956)	23	0.937 (0.908–0.970)	14	47	8	24	25
**Portugal**	36	17	0.951 (0.930–0.972)	22	0.956 (0.922–0.989)	11	28	8	10	26
**USA**	72	34	0.984 (0.976–0.985)	45	0.975 (0.961–0.988)	33	48	23	70	1
**—NYMC**	11	8	0.927 (0.833–1.020)	10	0.981 (0.936–1.020)	5	6	5	9	0
**—CDC**	61	33	0.983 (0.979–0.988)	37	0.972 (0.957–0.988)	28	42	18	61	1
**Other**	15	12	0.971 (0.951–1.001)	13	0.981 (0.951–1.011)	10	7	7	1	6
***Total***	*178*	*37*	0.961 (0.954–0.967)	*80*	*0.966 (0.955–0.976)*	*n.a.*	*131*	*46*	*106*	*59*

a *D*, Simpsons Index of Diversity.

b CI, Confidence Interval.

n.a., not applicable.

With the inclusion of previously published MLST data on 61 invasive SDSE isolates obtained from the USA [25], a total of 37 *emm* types are represented among a larger set of 178 isolates, which is used for the analyses in this report. The frequencies of the different *emm* types span a wide range; however, only 38% of the *emm* types account for the majority (74%) of the isolates.

Among the 178 SDSE isolates, the number of alleles identified for each housekeeping gene locus ranges from 10 for *gtr*, *murI* and *mutS*, to 22 for *xpt* (Table 2). The *gki*, *recP*, and *xpt* loci exhibit the highest level of nucleotide sequence diversity (π). The percentage of polymorphic nucleotide sites ranges from 2.7 (n = 12) for *murI*, to 10.1 (n = 50) for *gki*. A significant portion of the polymorphism observed in *gki* can be attributed to *gki12*, a highly divergent allele with greater similarity to GAS *gki* alleles than to SDSE alleles (Figure S1); when the divergent *gki12* allele is removed from the analysis, the nucleotide diversity of *gki* falls from 0.021 to 0.012, and the percentage of polymorphic sites drops from 10.1 to 5.4 (n = 27). The d_n_/d_s_ ratio is less than one for each of the seven housekeeping genes, consistent with stabilizing selection.

**Table 2:** Housekeeping genes used for MLST of SDSE.

Gene	ORF^a^	Size of partial gene	No. of alleles	No. of nucleotide variant positions (%)	No. of variant aa positions^b^	π	d_n_	d_s_	d_n_/d_s_
Glucose kinase (*gki*)	SDEG_1515	498	12	50 (10.1)	7	0.021	0.0035	0.0736	0.047
Glutamine transport protein (*gtr*)	SDEG_1494	450	10	15 (3.3)	7	0.010	0.0059	0.0248	0.240
Glutamate racemase (*murI*)	SDEG_0413	438	10	12 (2.7)	1	0.012	0.0068	0.0142	0.479
DNA mismatch repair protein (*mutS*)	SDEG_2091	405	10	27 (6.7)	7	0.016	0.0062	0.0480	0.129
Transketolase (*recP*)	SDEG_1735	459	20	37 (8.1)	3	0.034	0.0008	0.1472	0.006
Xanthine phosphoribosyl transferase (*xpt*)	SDEG_0895	450	22	38 (8.4)	10	0.021	0.0049	0.0736	0.0665
Acetoacetyl-coathioloase (*atoB*)	SDEG_1700	434	12	18 (4.1)	5	0.011	0.0035	0.0330	0.106

a Based on ORF number in the GGS_124 genome (Genbank number AP010935).

b aa, amino acid.

### Relationships among STs

The seven housekeeping alleles of each of the 178 isolates yield a total of 80 distinct allelic profiles, referred to as sequence types (STs). Of the 80 STs, 37 are newly identified in this study and 43 were previously defined by Ahmad et al [25]. A minority of STs (10%) account for a disproportionate number (∼50%) of the total SDSE isolates under evaluation. The vast majority of STs (62, or 77%) are represented by only one SDSE isolate. The most prevalent ST (ST15) is represented by 20 isolates. Eight STs, each represented by eight or more isolates, account for 51% of the 178 SDSE isolates characterized by MLST.

**Figure 1:**
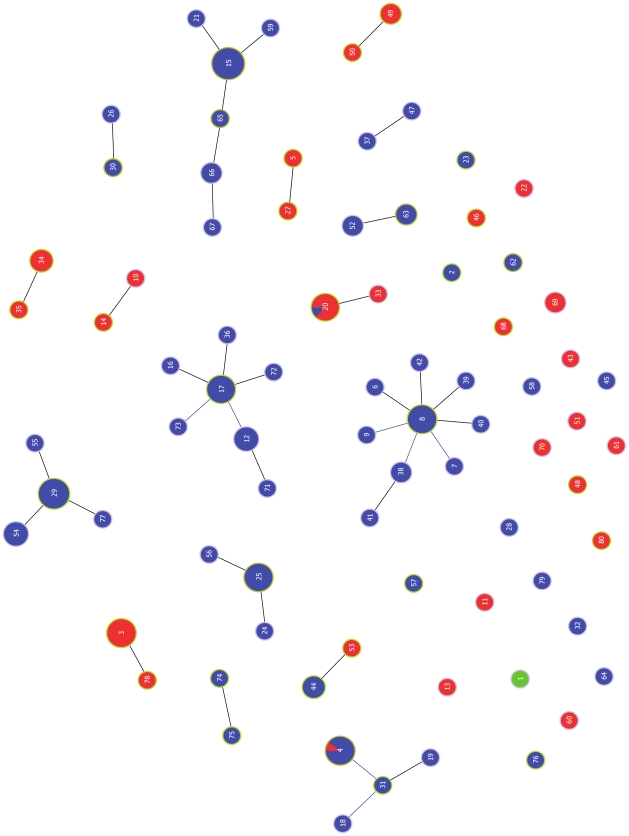
goeBURST diagram of relationships between 178 global SDSE isolates. The size of each circle is proportional to the number of isolates with that particular ST in a logarithmic scale. STs assigned to the same CC are linked by straight lines. Blue circles represent isolates that have the group G carbohydrate. Red circles represent isolates expressing the group C carbohydrate. Whenever isolates of the same ST have different group carbohydrates, the number of isolates bearing the same carbohydrate is proportional to the respective color. The green circle represents the single isolate expressing the group L carbohydrate.

The 80 STs can be grouped into 17 clonal complexes (CCs) by goeBURST, whereby the connected STs are single locus variants (SLVs) of at least one other ST in the group, differing at only one housekeeping gene (Figure 1). However, only six of the 17 CCs contain more than two STs. CC8 contains the highest number of STs (n = 9), whereas CC15 (n = 26) and CC29 (n = 20) contain the most isolates. Twenty-six STs, representing 15% of the 178 isolates, are singletons and differ from all other STs by more than two housekeeping alleles. When clusters are constructed linking STs that are up to triple locus variants (TLVs) of each other, 66 STs are grouped into a single major cluster, whereas only three STs remain ungrouped singletons (Figure S3), indicating that several intermediate genotypes probably exist, but have not yet been sampled.

### Relationships between *emm* type and ST

The overall correspondence between ST and *emm* type, as determined by the Wallace Coefficient, is low (ST vs *emm* type W = 0.473, CI_95%_ 0.332–0.542; *emm* type vs ST W = 0.384, CI_95%_ 0.311–0.456) reflecting the fact that most *emm* types are found in multiple STs, and that the same ST can harbor different *emm* types (Table 3 and Table 4). The correspondence between *emm* type and CC (W = 0.551, CI_95%_ 0.478 to 0.625) is also weak.

**Table 3:** Relationship between ST and emm type.

ST	No. of isolates	Associated *emm* types	No. of *emm* types
15	20	stC839, stG10, stG166b, stG2078, stG245, stG6, stG652	7
8	10	stC839, stG11, stG480, stG643, stG7860	5
4	10	stC36, stC5344, stG6792, stG97, stG7882	5
3	10	emm57, stC1400, stC839, stG653	4
25	9	stG166b, stG5420, stG6	3
17	9	stC74a, stG2078, stG485	3
63	2	stG6, stG652	2
52	2	stG6, stG643	2
20	8	stC6979, stG62647	2
34	3	stC1400, stG5063	2
29	14	stC74a, stG485	2

**Table 4:** Relationship between *emm* type and ST.

*emm* type	No. of isolates	Associated STs	No. of STs	No. of CC_SLV_ a	No. of CC_DLV_ b	No. of distant STs^c^
stG6	13	15, 24, 25, 44, 52, 58, 62, 63	8	4	2	2
stG480	11	7, 8, 38, 39, 40, 41, 67	7	2	2	2
stC1400	8	3, 28, 34, 46, 64, 66	6	3	1	4
stG485	8	17, 29, 37, 47, 55, 69	6	3	2	2
stG643	9	8, 12, 22, 48, 52, 73	6	3	4	5
stG652	7	15, 32, 59, 61, 63, 71	6	3	2	
stC6979	8	9, 19, 20, 54, 80	5	4	4	3
stC36	6	4, 45, 49, 50, 68	5	2	1	2
stC74a	15	17, 29, 70, 77	4	2	3	3
stC839	7	3, 8, 15, 78	4	3	2	3
stG166b	5	15, 25, 56, 65	4	2	2	2
stG11	5	6, 8, 42	3	1	1	
stG2078	9	15, 17, 72	3	2	2	2
stG245	3	15, 21, 36	3	2	2	2
stG4831	3	74, 75, 76	3	1	0	
stG62647	9	20, 33, 60	3	1	1	
stG6792	6	4, 31, 51	3	1	1	
emm57	3	3, 57	2	1	0	2
stC5344	3	4, 43	2	1	1	
stC6746	2	5, 27	2	1	0	
stC9431	2	13, 14	2	1	1	
stG5063	2	2, 34	2	1	0	
stG840	2	26, 30	2	1	1	
stG7882	2	4, 18	2	1	1	

a CC_SLV_ – Clonal complex based on Single Locus Variant relationships.

b CC_DLV_ – Clonal complex based on Double Locus Variant relationships.

c Number of STs sharing the same *emm* type and differing from all other STs harboring that *emm* type at greater than five housekeeping alleles.

STs associated with multiple *emm* types most likely arose via recombinational replacement of the *emm* gene; they are referred to as *emm* variable STs. Of the 18 STs represented by more than one isolate, 11 STs (61%) are associated with more than one *emm* type (Table 3). Five STs are associated with two *emm* types (ST34, 20, 29, 52, 63), two STs (ST17, 25) are associated with three *emm* types, one ST (ST3) is associated with four *emm* types, two STs (ST4, ST8) are associated with five *emm* types and one ST (ST15) is associated with 7 different *emm* types.

The same *emm* type is often found in association with multiple STs (Table 4). The association of a given *emm* type with multiple STs can arise following diversification of housekeeping genes, or by horizontal transfer of the *emm* gene. An estimate of the horizontal movement of *emm* is made by enumerating the number of distant STs harboring the same *emm* type, whereby distant STs are defined as having five or more housekeeping allele differences to any other ST that shares the same *emm* type; for a given CC, only one representative ST is counted.

Thirteen *emm* types are associated with distant STs: five *emm* types are associated with >two distant STs and eight *emm* types are found among a single pair of distant STs, whereas five *emm* types are associated with three or more distant STs (Table 4). The most promiscuous *emm* type is *emmstG643*, found among five genetically distant strains. A total of 21 horizontal transfer events involving *emm* genes are evident in the SDSE data set. Taken together, the data provides strong support for the hypothesis that *emm* genes of SDSE undergo extensive lateral exchange between strains.

### Relationships between group carbohydrate and ST

The group specific carbohydrate of the streptococcal cell wall can be used to discriminate among β-hemolytic streptococcal species. The majority of SDSE isolates (74%) in this study express the group G carbohydrate. A sharp distinction between STs associated with strains expressing the group G versus C carbohydrate is observed by goeBURST (Figure 1). Isolates representing 54 STs have group G carbohydrate (group G streptococci, GGS), whereas 27 STs are associated with strains expressing group C carbohydrate (group C streptococci; GCS). A single isolate has the group L carbohydrate.

Only two STs (ST4 and ST20) have isolates associated with both group C and G carbohydrates; for ST4, nine of 10 isolates are GGS and for ST20, seven of eight isolates are GCS. Overall, ST and group carbohydrate, whose biosynthesis locus is unknown, display very strong linkage (W = 0.970, CI_95%_ 0.939 to 1.000). The correspondence between CCs and group carbohydrate is also high, with nine CCs containing only GGS isolates and six CCs restricted to GCS isolates (W = 0.979, CI_95%_ 0.957 to 1.000), indicating that STs belonging to the same genetic lineage almost always also share the same group carbohydrate.

For each of the seven housekeeping loci, the relative distribution of alleles among GCS and GGS isolates was evaluated (Table 5). Overall, 38% of the housekeeping alleles are shared among GCS and GGS isolates. Approximately 36% and 29% of the housekeeping alleles are restricted to GCS and GGS isolates, respectively. This finding shows that there is a common housekeeping gene pool that is shared among numerous GCS and GGS isolates, despite the highly restricted associations that are observed between group carbohydrate and ST.

**Table 5:** Distribution of housekeeping alleles among GCS and GGS isolates.

Housekeeping gene locus^a^	% of alleles shared by GCS and GGS	% of alleles restricted to GCS	% of alleles restricted to GGS
*murI*	30	30	40
*xpt*	32	36	32
*gtr* b	56	33	11
*gki*	33	25	43
*atoB*	50	33	17
*recP*	35	35	30
*mutS*	40	30	30
Total for all alleles	38	36	29

a Presented in order of the locus position on the genome of strain GGS_124.

b Excludes *gtr06* which is restricted to group L.

### Relationships between group carbohydrate and *emm* type

Unlike ST, which is largely restricted to a single group carbohydrate form, 13 (35%) of the 37 *emm* types are found in association with both GGS and GCS isolates (W = 0.821, CI_95%_ 0.764 to 0.876). Eight of the variable associations between *emm* type and group carbohydrate likely arose following horizontal transfer of an *emm* gene to a strain having a different group carbohydrate (data not shown). However, it remains possible that lateral movement of genes encoding carbohydrate biosynthetic enzymes also contributes to the generation of diversity among SDSE, although the frequency of this event is probably low because carbohydrate-variable STs are rare.

### Geographic distribution of genetically diverse SDSE isolates

To examine the global distribution of SDSE clones, the ST and *emm* type of the isolates recovered from Australia, Europe and North America were compared. Clonal diversity based on either ST or *emm* type, as measured by the Simpson Index of Diversity, was high (>0.9) for isolates collected from each of the three primary locations (Table 1).

**Figure 2:**
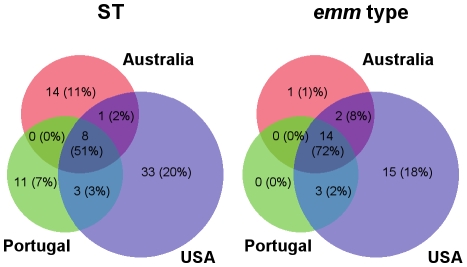
Venn diagram depicting the distribution of ST and *emm* type across three continents. Unbracketed numbers represent the total number of STs or *emm* types. The numbers in brackets indicate the percentage of total isolates in the entire collection.

The eight STs that were recovered from all three continents also represent the STs with the highest overall prevalence (ST3, 4, 8, 15, 17, 20, 25, 29) (Table S1; Figure 2). Furthermore, five of the eight highly prevalent STs are predicted to be founders of a CC, wherein the founder ST is defined as that having the highest number of SLVs. The data suggest a wide geographical dispersion of founder STs. Of the next 10 most highly prevalent STs (each of which is represented by two to four isolates), four were associated with isolates from two continents (Table S1). Fourteen *emm* types were recovered from each of the three continents; 13 of these are represented by the most highly prevalent strains, having greater than 5 SDSE isolates per *emm* type. Together the results demonstrate that the most highly prevalent strains of SDSE, whereby strain is defined by either ST or *emm* type, are widely disseminated.

Among the 178 SDSE isolates, 107 unique combinations of ST, *emm* type, and group carbohydrate were observed. Isolates corresponding to eight of the 107 unique *emm* ST-carbohydrate profiles are widespread and were recovered from all three continents (Table 6); another seven were isolated from two continents. Three intercontinental clones belong to CC8, two of which likely arose from a common ancestor by either genetic diversification at a housekeeping gene locus and/or by horizontal transfer of the *emm* gene. The two intercontinental CC17 clones are SLVs, whereas the CC15 and CC25 sets of intercontinental strains arose via lateral exchange of *emm* type. The genetic changes are likely to be ancient events that preceded the intercontinental migration of the founders.

**Table 6:** Intercontinenal clones of SDSE.

CC	ST	*emm* type	group carbohydrate	Australia	Europe	North America
3	3	stC839	C	x	x	x
4	4	stG6792	G		x	x
8	8	stG480	G	x	x	x
8	8	stG11	G	x		x
8	38	stG480	G	x	x	x
15	15	stG10	G	x	x	x
15	15	stG652	G	x		x
15	15	stG166b	G		x	x
17	17	stG2078	G	x	x	x
17	12	stG643	G	x		x
20	20	stG62647	C	x	x	x
25	25	stG5420	G	x	x	x
25	25	stG6	G	x	x	
29	29	stC74a	G	x	x	x
49	49	stC36	C		x	x

### Phylogenetic analysis of housekeeping genes

Clonal relationships established via goeBURST are based on the character state of the housekeeping gene allele, and do not take into account the degree of nucleotide sequence heterogeneity. In order to further investigate the relatedness of the housekeeping gene alleles at each locus, phylogenetic trees for each gene were constructed by the neighbor joining method. With the exception of *atoB*, these trees included the alleles from loci of GAS having the highest percentage nucleotide sequence identity based on BLASTn. Additionally, the housekeeping genes of GAS and SDSE share synteny (Table 2) [20]. In agreement with a previous report [25], several SDSE alleles are more similar to GAS alleles than to other SDSE alleles (Figures S1, S2). Both *gki12* and *mutS3* form a cluster with GAS alleles, whereas all *gtr* and *murI* alleles from SDSE and GAS segregate into distinct species-specific clusters. The relationship between *recP* and *xpt* alleles in the two species is more complex, and the phylogenies for the *xpt* and *recP* alleles do not resolve into species-specific clusters.

**Figure 3:**
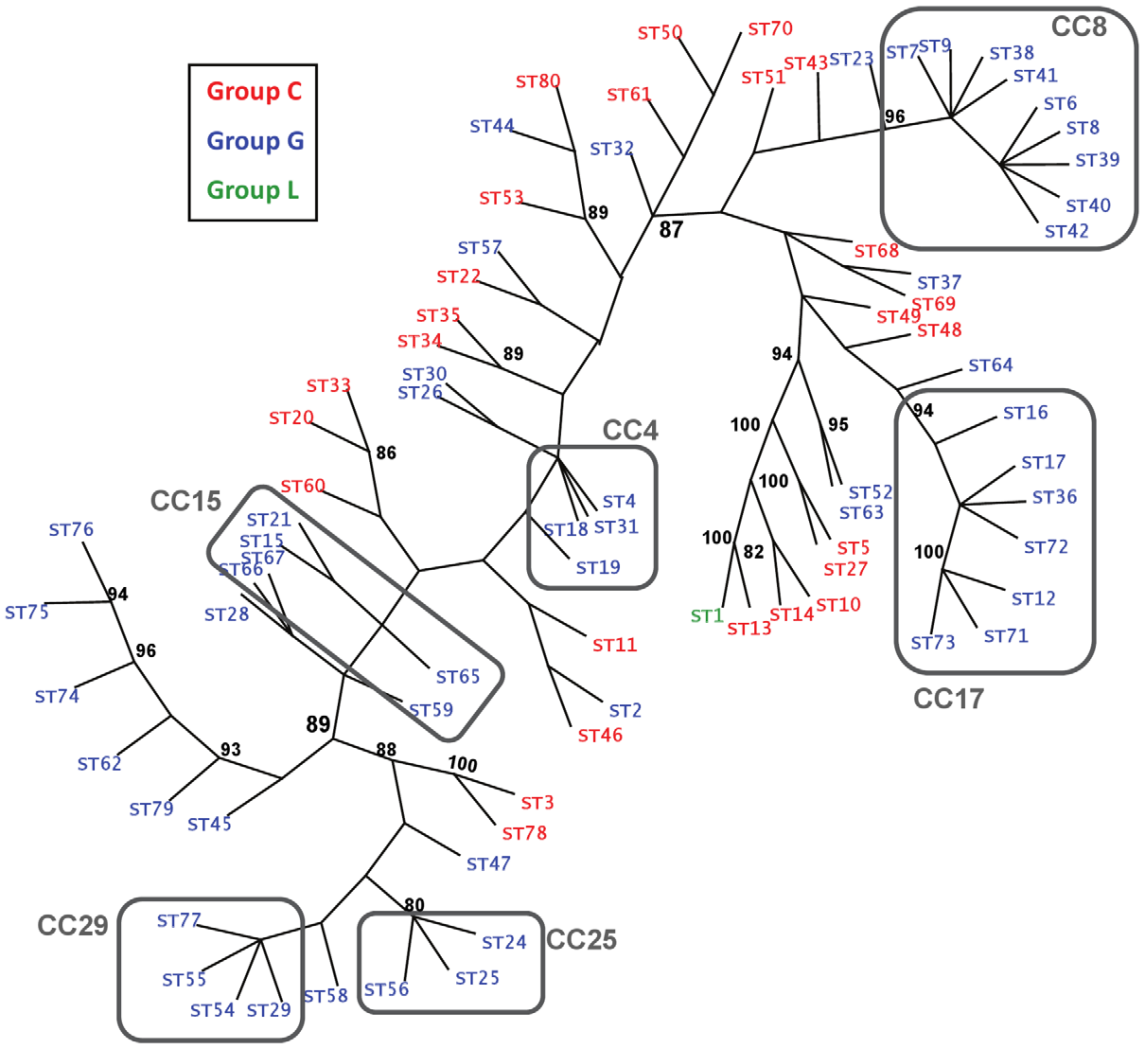
Maximum parsimony tree of concatenated housekeeping alleles. The housekeeping alleles for each of the 80 STs for SDSE were concatenated (3,134 nt positions), and a maximum parsimony tree was constructed. The radial, unrooted phylogenetic tree is shown. Bootstrap values (500 replicates) showing branch support equal or greater than 80% are indicated; bootstrap analysis used a heuristic search and the 50% majority-rule consensus tree is presented. STs representing GCS and GGS are depicted in red and blue, respectively; the single group L isolate (ST1) is depicted in green. CCs having three or more STs are indicated. Characters: 2937 are constant, 56 variable characters are parsimony-uninformative, 141 are parsimony-informative. Consistency index (CI)  =  0.3350; CI excluding uninformative characters  =  0.2669; retention index (RI)  =  0.7926.

The seven housekeeping alleles were concatenated (3,134 nucleotide sites) for each of the 80 STs of SDSE, and the concatenates used to construct a phylogenetic tree by the maximum parsimony method (Figure 3). The relative distribution of STs along the tree branches is highly concordant with the CCs generated via the goeBURST clustering algorithm that used allele character states (Figure 1). A striking feature of the phylogenetic tree is that GCS and GGS taxa are highly interspersed and fail to form discrete evolutionary lineages, even in portions of the tree having strong bootstrap support. However, the homoplasy index for the phylogenetic tree is high (0.7331, excluding uninformative characters) and strong bootstrap support is absent for many of the deeper branches. Thus, the phylogeny may be less accurate for long term evolutionary events due to a past history of extensive recombination, but nevertheless, it appears to recapitulate the short term evolution detected by goeBURST. The analysis also provides additional support for the horizontal transfer of housekeeping genes between GCS and GGS organisms.

Phylogenetic trees constructed by the minimum evolution (data not shown) or neighbor joining (Figure S4) methods also exhibit high concordance with the CCs generated via goeBURST. The only exception is CC15, which is divided into two or three small subclusters. However, like the maximum parsimony tree (Figure 3), there is little bootstrap support for deep branches in the neighbor joining tree (Figure S4).

### Role of recombination in genetic change in SDSE

An analysis of recombination events between the GAS and SDSE housekeeping gene alleles, using the Recombination Detection Program (RDP) suite [26], predicts extensive recombination between GAS and SDSE in the *gki* gene. The *gki75* and *gki102* alleles of GAS were identified as having derived from recombination with SDSE alleles (p<0.00001 and p = 0.00037, respectively), whereas the *gki12* and *gki4* of SDSE appear to result from recombination with GAS alleles (both with p<0.00001). These data account for the high nucleotide percentage diversity observed in the *gki* locus. Recombination between GAS and SDSE could also be identified using RDP in the *recP* gene. The GAS *recP21*, *recP40*, *recP54*, *recP71*, *recP85* are presumed to have resulted from recombination with SDSE alleles (p = 0.02469, p = 0.01073, p = 0.00050, p = 0.00900 and p = 0.00900, respectively). In contrast, SDSE alleles *recP12* and *recP3* seem to result from recombination with GAS alleles (p = 0.00262 and p = 0.00231, respectively). No significant recombination events between the other GAS and SDSE housekeeping alleles (*gtr*, *murI*, *mutS*, *xpt*) were observed using RDP. The SplitsTrees analysis for networked evolution was also used to assess intragenic recombination involving the MLST genes within the SDSE population. Statistically significant evidence for recombination (PHI test) was observed for *murI*, *recP*, *xpt* and *atoB* (p<0.01).

It is important to note that the recombination detection algorithms do not detect the complete replacement of the analyzed fragment. As pointed out previously, at least one instance of recombination involving the entire fragment of *mutS* analyzed is also suggested by phylogenetic analysis (Figure S2). Taken together, these data strongly suggest that intra- and inter-species recombination has occurred for several housekeeping genes.

The largest CC (CC8) contains 11.3% of the total STs, and is within the range of reliable performance of the BURST rules [27]. To estimate the relative role of recombination versus mutation in the short-term evolution of SDSE, the SLVs linked by goeBURST were examined for genetic differences between the variant alleles of each SLV pair. goeBURST identified 38 primary SLVs. Of these, 31 are predicted to have arisen via recombination, and seven SLVs are predicted to arise through point mutation. Thus, recombination occurred 4.4 times more often than mutation. The per site recombination to mutation (*r/m*) ratio was 20.7. An additional four SLVs, representing alternative ST relationships, are also present in the data set; of these, three SLVs are predicted to arise through recombination. The empirical findings on the relative contribution of recombination versus mutation to the genetic diversification of housekeeping genes are highly consistent with other findings that indicate extensive recombination involving SDSE.

## Discussion

*S. dysgalactiae* subsp *equisimilis* is increasingly recognized as an important human pathogen that causes disease in many regions of the world. The findings of this report demonstrate that the major genotypes of SDSE have an intercontinental distribution. The recovery from all three continents of the likely founder ST of at least five CCs supports a model whereby a few successful clones have undergone extensive migration, followed by genetic diversification. Several of the descendents are also widely disseminated, indicative of subsequent waves of clonal migration.

SDSE is largely a commensal species, yet the vast majority of isolates evaluated in this study were recovered from cases of human disease. The mode of person-to-person transmission of SDSE has not been well-characterized, and there may be large differences among SDSE strains in terms of their virulence properties and ease of transmission to new hosts. It stands to reason that the most widely disseminated clones are probably among the most readily transmitted. Whether transmission is positively linked to virulence is an important question that remains to be established for SDSE. Molecular typing of SDSE, as provided in this report, provides a framework upon which the question of whether or not subpopulations of SDSE strains have heightened virulence can be addressed.

More than half (60%) of the SDSE isolates studied have a unique combination of *emm* type, ST and group carbohydrate, indicative of a very high level of genetic diversity within the species. The high level of strain diversity may be a consequence of a high rate of genetic change and/or a very large population size. Nonetheless, molecular typing using only the *emm* gene versus MLST, yields stratifications that are highly discordant, and neither method by itself is satisfactory for defining strains or clones. This finding provides support for a role of extensive horizontal gene transfer and recombination in promoting random associations of *emm* and ST.

Recombination following horizontal gene transfer in SDSE is observed at several levels of biological importance, involving core housekeeping genes and the *emm* gene, and perhaps even the genes encoding the group carbohydrate biosynthetic enzymes, albeit at a much lower frequency. Inter-specific gene transfer between SDSE and GAS is likely for several of the housekeeping genes. Interestingly, ST3 and ST78 isolates possess the SDSE *recP6* and *xpt2* alleles, which are identical to the GAS derived *recP83* and *xpt4* alleles respectively. Although it is formally possible that the *recP* and xpt alleles were transferred in a single genetic exchange event involving a large genome segment, this hypothesis is unlikely because SDSE-like *gki* and *gtr* alleles are positioned in between the *xpt* and *recP* loci on the SDSE genome.

Transfer of *emm* genes between SDSE and GAS also seem likely to have occurred, but to a much more limited extent, as evidenced only by the prototypical GAS *emm* types *emm57* [25] and *emm12* [28]. In addition to *emm* and the housekeeping genes, lateral exchange between GAS and SDSE is documented for several other genes, including those encoding a fibronectin-binding protein [29], a DNA gyrase subunit implicated in fluoroquinolone resistance [30], a transcriptional regulator of pilus gene expression [31], and the plasminogen activator streptokinase [32], [33]. Thus, SDSE and GAS share common gene pools for numerous loci.

Intra-species genetic exchange among SDSE organisms, involving either the core housekeeping genes or the *emm* gene, has been extensive. Data suggest recombination to be the predominant mechanism of genetic diversification among SDSE, occurring four times more often than point mutation in housekeeping loci. The per-site recombination to mutation ratio is also greater than 20. More than half of the STs represented by >one isolate are considered to be *emm* variable STs, and are found in association with at least two distinct *emm* types. ST15 is a particularly successful recipient of *emm* genes originating from multiple SDSE donor strains, as evidenced by its recovery in association with seven different *emm* types. Mechanisms that might explain the existence of STs having associations with many distinct *emm* types include possession of genetic machinery that increases their probability for recombinational success, additional accessory genes which facilitate their transmission to new hosts, a high prevalence (eg., ST15 comprises 20 of the 178 isolates) or natural selection favoring the emergence of variants harboring unique *emm* types.

Numerous *emm* gene donor-recipient ST pairs are also represented within the SDSE isolate data set, wherein 13 different *emm* types are associated with two or more genetically distant STs; in total, 21 distinct *emm* gene horizontal transfer events are suggested by the data. One *emm* type (*stG643*) is found in association with five distant STs or CCs. The *emm* gene of GAS is part of an ancient pathogenicity island [34]. Thus, it will be of interest to determine whether the *stG643* gene is harbored by a functional mobile genetic element.

In GAS, the *emm* gene product (M protein) is a major virulence factor and a primary target of protective immunity by the human host. However, to our knowledge, it remains to be shown that the M protein of SDSE prevents phagocytosis in the absence of M type-specific antibody, which is a hallmark feature of GAS. Thus, the relationship between gene replacements involving the *emm* gene, and positive selection arising from host immune pressures, remains speculative for SDSE. The M proteins of GAS are also multifunctional proteins, which as a group, exhibits binding for numerous host proteins that include plasminogen, fibrinogen, immunoglobulins and complement regulatory proteins, as recently reviewed by Smeesters et al [35]. Like GAS, M proteins of SDSE that can bind plasminogen have been identified [36]. However, the full extent by which the M proteins of SDSE share functional attributes with the M protein of GAS remains to be established. The acquisition of new, M protein-mediated functional activities by a recipient SDSE strain could conceivably drive selection for the emergence of novel *emm*-ST combinations among SDSE.

Group carbohydrate is synthesized by biosynthetic enzymes whose genes have yet to be characterized. Among SDSE, the association between group C versus G carbohydrate and ST approaches 100% concordance. The observed linkage between group carbohydrate and ST is probably a large reflection of short term evolution. An evolutionary history of housekeeping gene exchange between GCS and GGS is evident from the phylogenetic tree of concatenated housekeeping gene sequences. While it is likely that GCS and GGS diverged from a common ancestor, subsequent genetic exchange masks that history, making it difficult to ascertain the extent to which GCS and GGS comprise distinct evolutionary lineages. However, MLST data makes it clear that lateral gene transfer leading to a group carbohydrate switch is a rare event.

The goeBURST population snapshot of SDSE (Figure 2) differs from that reported for GAS [16]. For GAS, only 2.8% of the STs are present in the largest CC, as compared to 11.2% of the SDSE STs (i.e., CC8). Based on simulated bacterial populations differing in levels of recombination and diversity generated by mutation [27], the population genetic structure of GAS is best explained by high rates of both recombination and mutation acting on a diverse set of housekeeping genes. For SDSE, the recombination rate and housekeeping gene diversity may be similarly high; however the mutation rate may be somewhat lower than in GAS. This proposed genetic structure for the SDSE population is supported by correspondence to simulated populations [27], combined with findings that show a greater than 4-fold excess of recombinational events in the diversification of SLVs.

SDSE appears to be among the closest extant relatives of GAS. In general terms, a pathogenic species of bacteria can arise from an organism of lower virulence following acquisition of virulence genes (eg., pathogenicity islands). However, evolution can also flow in the opposite direction, as recently evidenced by the loss of virulence genes and descent of the commensal *Streptococcus mitis* from the pathogen *Streptococcus pneumoniae* [37]. Determination of whether the most recent common ancestor of GAS and SDSE is more closely related to GAS or to SDSE, will probably require more extensive genomic analyses. The molecular typing and characterization of the population biology of SDSE in this report provides a foundation for future studies that address the evolution and molecular basis for virulence in SDSE.

## Materials and Methods

### Bacterial Strains

The 117 SDSE isolates analyzed in this study were collected from Australia (n = 55), Portugal (n = 36) and the USA (n = 11). Another 15 SDSE isolates were obtained from other countries or had no associated geographic information (Table S1). All isolates were classified as SDSE on the basis of isolation from a human host, β-hemolysis following growth on sheep blood-containing agar, the presence of group C or G carbohydrate, and the presence of a recognized *emm* type. *emm* type was determined by nucleotide sequence typing as described [38], and *emm* type was assigned using the BLASTn-*emm* server (http://www.cdc.gov/ncidod/biotech/strep/strepblast.htm). Group carbohydrate was determined using the latex bead agglutination test. Forty-five isolates were obtained from normally sterile tissue or fluid and classified as causing invasive disease; 59 isolates were recovered from other non-sterile sites and classified as causing non-invasive infections (Table 1). Information on the 61 invasive isolates recovered from the USA, and reported in a previous study [25], was integrated into the current analyses to obtain a global overview of the relationships between GGS and GCS causing infections in humans.

### MLST

Seven housekeeping genes are used for MLST of SDSE. With the exception of *atoB*, the housekeeping genes used for MLST of SDSE are the same as those used in the GAS MLST scheme [38]. The seventh allele, *atoB*, (also called *yqi*Z) has been described [25]; the allele name has been changed for this report, in order to reduce confusion with the GAS MLST gene, *yqiL*, which occupies a different locus Primer sequences used for PCR amplification are listed in Table S2. Although our MLST scheme was developed independently from that reported previously [25], the data is directly comparable because the same genes, and regions within genes, are targeted for sequencing. All PCR products were sequenced in both the forward and reverse directions; newly identified alleles and alleles defining new STs were re-sequenced in order to validate the initial findings. Unique alleles at each locus were assigned individual allele numbers. The combination of the seven allele numbers for each isolate was used to define the sequence type (ST). MLST data for SDSE is available at www.mlst.net (pending publication).

### Data analysis

goeBURST (http://goeburst.phyloviz.net/) [39], which uses the same clustering rules as eBURST [40] but provides a global optimal solution, was used to determine the relationships between STs. Clonal complexes (CCs) are defined as STs that are linked through single locus variants (SLVs) and are named on the basis of the predicted founder ST, which is the ST having the most SLVs. In cases where a CC contains only two STs, the lower numbered ST was used to define the CC. Isolates that share four of seven alleles (i.e. triple locus variants, TLVs) were used to define larger, more distantly related clonal groups.

The Simpson Index of Diversity (*D*) and Wallace coefficients (W) were calculated as described previously [41] using www.comparingpartitions.info. A *D* value equal to one signifies that the typing method distinguishes between all isolates, whereas a *D* value equal to zero means that all isolates are identical. The W coefficient provides a finer comparison between two typing methods, since the value indicates the probability that two strains classified as the same type by one method are also classified as the same type by the other method. A high value of the W coefficient (values close to 1) indicates that partitions defined by a given method could have been predicted from the results of another method, suggesting that the use of both methodologies could be redundant. Nucleotide diversity (π), nonsynonymous (d_n_) and synonymous substitution rates (d_s_) were calculated using DnaSP (version 5) [42].

### Phylogenetic analysis

Phylogenetic relationships amongst individual housekeeping alleles was examined using the neighbor joining (NJ) method with Jukes-Cantor substitution algorithm model as implemented in MEGA4 [43]; bootstrapping (1000 replicates) was used to ascertain support for branches. For concatenated housekeeping alleles, evolutionary history was inferred using the NJ method or maximum parsimony method (PAUP 4.0).

### Recombination and point mutation

Empirical estimates of the number of mutation and recombination events contributing to the diversification of SLVs were made as previously described [21], [44], with additional modifications. The nucleotide sequence differences in the mismatched allele among the pair of STs that define an SLV are scored as genetic changes that arise due to either mutation or recombination. Nucleotide differences between the two variant alleles at greater than 1 nucleotide site is scored as a probable recombination event. Single nt differences between the two variant alleles that do not occur in other any other alleles among the SDSE set of strains are scored as a change likely due to point mutation. If the single nucleotide polymorphism is present in two or more alleles assigned to different CCs, the genetic change is scored as likely due to recombination. The relative ratio of recombination events versus mutation events was then determined. To calculate the per site recombination/mutation (*r*/*m*) ratio, the total number of nucleotide sites that change due to recombination were divided by the total number of nucleotide sites that change due to mutation.

Recombination among SDSE and between the SDSE and GAS at each individual gene was evaluated using RDP [26], which implements a large number of methods (RDP, GENECONV, MaxChi, Chimaera, SiScan, 3Seq) for detecting intragenic recombination. For these analyses the entire collection of GAS alleles was downloaded from the MLST database (http://spyogenes.mlst.net). Since a high number of comparisons were performed, the p values reported are corrected for multiple tests. SplitsTrees4 was also used to assess intragenic recombination [45], excluding parsimony uninformative sites, and using the neighbor net algorithm, uncorrected P distance, and the Phi test for recombination [46].

## Supporting Information

Table S1 Characteristics of SDSE isolates in this study.
(0.25 MB DOC)

Table S2 PCR primer pairs used for MLST in the study.
(0.03 MB PDF)

Figure S1 Evolutionary history of gki, gtr, murI and atoB alleles from SDSE and GAS. Evolutionary relationships were inferred using the Neighbour-Joining (NJ) method, and evolutionary distances calculated using the the Jukes-Cantor method. Branches with bootstrap support (n = 1000) greater than 80% are shown next to their respective branches. The tree is drawn to scale, with branch lengths in the same units as those of the evolutionary distances used to infer the phylogenetic tree. Only names of the GGS alleles are shown. Phylogenetic analyses were conducted in MEGA4.
(0.12 MB TIF)

Figure S2 Evolutionary history of mutS, xpt and recP alleles from SDSE and GAS. Evolutionary relationships were inferred using the Neighbour-Joining (NJ) method, and evolutionary distances calculated using the the Jukes-Cantor method. Branches with bootstrap support (n = 1000) greater than 80% are shown next to their respective branches. The tree is drawn to scale, with branch lengths in the same units as those of the evolutionary distances used to infer the phylogenetic tree. Only names of the GGS alleles are shown. Phylogenetic analyses were conducted in MEGA4.
(0.02 MB PDF)

Figure S3 goeBURST diagram of the relationships between 178 global SDSE isolates grouped up to TLV. The size of each circle is proportional to the number of isolates with that particular ST in a logarithmic scale. STs differing up to three alleles (triple-locus variants - TLVs) are linked by straight lines. Black lines link STs differing at a single gene. Intermediate grey lines link STs differing at a two genes. Light grey lines link STs differing at a three genes. Blue circles represent isolates that have the group G carbohydrate. Red circles represent isolates expressing the group C carbohydrate. Whenever isolates of the same ST have different group carbohydrates, the number of isolates bearing the same carbohydrate is proportional to the respective color. The green circle represents the single isolate expressing the group L carbohydrate. The proposed founders of particular clusters are indicated by a light green outer circle. The sub-founders (defined has having links to three or more STs) are indicated by dark green outer circles.
(0.02 MB PDF)

Figure S4 Neighbor joining tree of concatenated housekeeping alleles. The housekeeping alleles for each of the 80 STs for SDSE were concatenated (3,134 nt positions), and a neighbor joining tree was constructed (MEGA4). Bootstrap values (1,000 replicates) showing branch support equal or greater than 80% are indicated STs representing GCS and GGS are depicted in red and blue, respectively; the single group L isolate (ST1) is depicted in green. CCs having three or more STs are indicated.
(0.06 MB PDF)
