# Supplementary material for: Population Genetics of Streptococcus dysgalactiae Subspecies equisimilis Reveals Widely Dispersed Clones and Extensive Recombination
Source: PLoS One. 2010 Jul 23;5(7):e11741. doi: 10.1371/journal.pone.0011741 (PMC2909212; doi:10.1371/journal.pone.0011741)
Supplement: Table S1 — Characteristics of SDSE isolates in this study. (0.25 MB DOC) [file pone.0011741.s001.doc]

**Table S1**: Characteristics of SDSE isolates in this study.

| **Strain** | ***emm* type** | **Group carbohydrate** | **ST** | **Clonal Complex ^a^** | **Location** | **Collection** |
| --- | --- | --- | --- | --- | --- | --- |
| 168554 | stG485 | G | 47 | 37 | Portugal | UL |
| 171712 | stG480 | G | 38 | 8 | Portugal | UL |
| 220269 | stG2078 | G | 15 | 15 | Portugal | UL |
| 223754 | stC839 | C | 3 | 3 | Portugal | UL |
| 230631 | stG480 | G | 8 | 8 | Portugal | UL |
| 231995 | stC74a | G | 29 | 29 | Portugal | UL |
| 241940 | stC36 | C | 50 | 49 | Portugal | UL |
| 273600 | stG166b | G | 65 | 15 | Portugal | UL |
| 299298 | stG643 | G | 8 | 8 | Portugal | UL |
| 313247 | stG6 | G | 25 | 25 | Portugal | UL |
| 363962 | stG2078 | G | 17 | 17 | Portugal | UL |
| 378119 | stC839 | G | 15 | 15 | Portugal | UL |
| 380870 | stG480 | G | 41 | 8 | Portugal | UL |
| 386041 | stC839 | C | 3 | 3 | Portugal | UL |
| 394314 | stG2078 | G | 72 | 17 | Portugal | UL |
| 423738 | stG62647 | C | 20 | 20 | Portugal | UL |
| 450784 | stG10 | G | 15 | 15 | Portugal | UL |
| 460880 | stG10 | G | 15 | 15 | Portugal | UL |
| 493188 | stG485 | C | 69 | s | Portugal | UL |
| 542567 | stG6 | G | 62 | s | Portugal | UL |
| 618280 | emm57 | G | 57 | s | Portugal | UL |
| SH0004 | stG6792 | G | 4 | 31 | Portugal | UL |
| SH0015 | stG6 | G | 25 | 25 | Portugal | UL |
| SH0032 | stG166b | G | 15 | 15 | Portugal | UL |
| SH0102 | stG2078 | G | 17 | 17 | Portugal | UL |
| SH0107 | stG643 | G | 52 | 52 | Portugal | UL |
| SH0110 | stG6 | G | 25 | 25 | Portugal | UL |
| SH0113 | stG6792 | G | 4 | 31 | Portugal | UL |
| SH0124 | stG6792 | G | 4 | 31 | Portugal | UL |
| SH0218 | stG245 | G | 15 | 15 | Portugal | UL |
| SH0254 | stG485 | C | 69 | s | Portugal | UL |
| SH0257 | stC6979 | C | 80 | s | Portugal | UL |
| SH0259 | stG652 | G | 71 | 17 | Portugal | UL |
| SH0275 | stG485 | G | 55 | 29 | Portugal | UL |
| SH0330 | stC36 | C | 49 | 49 | Portugal | UL |
| SH0336 | stG5420 | G | 25 | 25 | Portugal | UL |
| G121 | stC74a | G | 29 | 29 | Australia | QIMR |
| G122 | stC74a | G | 29 | 29 | Australia | QIMR |
| GCS10128 | stC1400 | C | 46 | s | Australia | QIMR |
| GCS2816 | stG62647 | C | 20 | 20 | Australia | QIMR |
| GCS6894 | stG62647 | C | 20 | 20 | Australia | QIMR |
| GCS6929 | stG62647 | C | 20 | 20 | Australia | QIMR |
| GGS075 | stG166b | G | 56 | 25 | Australia | QIMR |
| GGS101 | stG643 | G | 12 | 17 | Australia | QIMR |
| GGS10b | stG6 | G | 44 | 44 | Australia | QIMR |
| GGS11172 | stC74a | G | 29 | 29 | Australia | QIMR |
| GGS11543 | stG643 | G | 12 | 17 | Australia | QIMR |
| GGS120 | stG4831 | G | 74 | 74 | Australia | QIMR |
| GGS19 | stC1400 | G | 64 | s | Australia | QIMR |
| GGS2 | stG10 | G | 15 | 15 | Australia | QIMR |
| GGS24 | stG6 | G | 44 | 44 | Australia | QIMR |
| GGS430 | stG643 | G | 12 | 17 | Australia | QIMR |
| GGS463 | stG10 | G | 15 | 15 | Australia | QIMR |
| GGS539813 | stC74a | G | 29 | 29 | Australia | QIMR |
| GGS540048 | stG485 | G | 29 | 29 | Australia | QIMR |
| GGS545448 | stG10 | G | 15 | 15 | Australia | QIMR |
| GGS569 | stC6979 | G | 54 | 29 | Australia | QIMR |
| GGS592 | stG480 | G | 8 | 8 | Australia | QIMR |
| **Strain *cont*** | ***emm* type** | **Group carbohydrate** | **ST** | **Clonal Complex** | **Location** | **Collection** |
| GGS9225 | stC74a | G | 29 | 29 | Australia | QIMR |
| GGS985 | stC1400 | G | 66 | 15 | Australia | QIMR |
| GGSRHD | stG480 | G | 39 | 8 | Australia | QIMR |
| MD01 | stG11 | G | 8 | 8 | Australia | QIMR |
| MD02 | stG10 | G | 15 | 15 | Australia | QIMR |
| MD03 | stG2078 | G | 17 | 17 | Australia | QIMR |
| MD031 | stC74a | G | 17 | 17 | Australia | QIMR |
| MD04 | stG6 | G | 25 | 25 | Australia | QIMR |
| MD05 | stG6 | G | 63 | 52 | Australia | QIMR |
| MD06 | stC74a | G | 29 | 29 | Australia | QIMR |
| MD07 | stG6 | G | 58 | s | Australia | QIMR |
| MD08 | stG2078 | G | 17 | 17 | Australia | QIMR |
| MD09 | stG10 | G | 15 | 15 | Australia | QIMR |
| MD122 | stC74a | G | 29 | 29 | Australia | QIMR |
| MD136 | stG10 | G | 15 | 15 | Australia | QIMR |
| MD163 | stG652 | G | 63 | 52 | Australia | QIMR |
| MD227 | stC6979 | C | 20 | 20 | Australia | QIMR |
| MD248 | stC6979 | G | 54 | 29 | Australia | QIMR |
| MD296 | stG10 | G | 15 | 15 | Australia | QIMR |
| MD411 | stC1400 | G | 66 | 15 | Australia | QIMR |
| MD499 | stC6979 | G | 54 | 29 | Australia | QIMR |
| MD504 | stC839 | C | 3 | 3 | Australia | QIMR |
| MD605 | stG62647 | C | 20 | 20 | Australia | QIMR |
| MD633 | stG643 | G | 73 | 17 | Australia | QIMR |
| MD722 | stC74a | G | 29 | 29 | Australia | QIMR |
| MD805 | stG652 | G | 59 | 15 | Australia | QIMR |
| MD834 | stC36 | G | 4 | 31 | Australia | QIMR |
| MD872 | stG62647 | C | 60 | s | Australia | QIMR |
| MD921 | stC6979 | G | 54 | 29 | Australia | QIMR |
| MD934 | stG5420 | G | 25 | 25 | Australia | QIMR |
| NS1121 | stG4831 | G | 75 | 74 | Australia | QIMR |
| NS542 | stG652 | G | 15 | 15 | Australia | QIMR |
| NS752 | stG6 | G | 44 | 44 | Australia | QIMR |
| GCS01ny | stC1400 | C | 3 | 3 | Other | NYMC |
| GCS04ny | stC5344 | C | 43 | s | Other | NYMC |
| GCS07ny | stG6792 | C | 51 | s | Other | NYMC |
| GCS08ny | stC5345 | C | 53 | 44 | Other | NYMC |
| GCS10ny | stC74a | C | 70 | s | Other | NYMC |
| GCS11ny | stC839 | C | 78 | 3 | Other | NYMC |
| GGS12ny | stG11 | G | 8 | 8 | Other | NYMC |
| GGS13ny | stG480 | G | 8 | 8 | Other | NYMC |
| GGS15ny | stC74a | G | 29 | 29 | Other | NYMC |
| GGS16ny | stG485 | G | 29 | 29 | Other | NYMC |
| GGS19ny | stG11 | G | 42 | 8 | Other | NYMC |
| GGS24ny | stG4974 | G | 79 | s | Other | NYMC |
| GGS22ny | stG4831 | G | 76 | s | Other | NYMC |
| GGS 124 | stG480 | G | 67 | 15 | Other | other |
| DSM6176 | stG652 | C | 61 | s | Other | other |
| GCS02ny | stC839 | C | 3 | 3 | USA | NYMC |
| GCS03ny | stG653 | C | 3 | 3 | USA | NYMC |
| GCS05ny | stG643 | C | 48 | s | USA | NYMC |
| GCS06ny | stC36 | C | 49 | 49 | USA | NYMC |
| GCS09ny | stC36 | C | 68 | s | USA | NYMC |
| GGS14ny | stG10 | G | 15 | 15 | USA | NYMC |
| GGS17ny | stG480 | G | 38 | 8 | USA | NYMC |
| GGS18ny | stG480 | G | 40 | 8 | USA | NYMC |
| GGS20ny | stC36 | G | 45 | s | USA | NYMC |
| GGS21ny | stG6 | G | 52 | 52 | USA | NYMC |
| GGS23ny | stC74a | G | 77 | 29 | USA | NYMC |

^a^ ‘s’ denotes singleton
