# Supplementary material for: Population Genetics of Streptococcus dysgalactiae Subspecies equisimilis Reveals Widely Dispersed Clones and Extensive Recombination
Source: PLoS One. 2010 Jul 23;5(7):e11741. doi: 10.1371/journal.pone.0011741 (PMC2909212; doi:10.1371/journal.pone.0011741)
Supplement: Table S2 — PCR primer pairs used for MLST in the study. (0.03 MB PDF) [file pone.0011741.s002.pdf]

Table S2. PCR primer pairs used for MLST in the study.

Locus	Forward primer	Forward primer sequence (5' to 3')	Reverse primer	Reverse primer sequence (5' to 3')	Nucleotide location in GGS_124[a]	Primers used by[b]
gki	Gkigc-up	GGAATTGGTATGGGATCACCAGGAGC	Gkigc-dn	AATTCTCCTGCTGCTGACAC	1473922-1473425	UL, NYMC, QIMR
gtr	Gtrgc-up	GCACAAGTATTATGGGCACA	Gtrgc-dn	CACGGTCTGCGACTTC	1450465-1450016	UL, QIMR
	Gtrgc-up2	AAATATTATGGGCAAAACGAGGTG	Gtrgc-dn2	CTTCCACAATAACGCCGCCATCCATA		NYMC
murI	MurIgc-up	GACCTGCTGAGCAAATTAGAGAATACA CATGGG	MurIgc-dn	CAGGACTTGCCGTTGTGTAAAAATGGTG	389637-390074	UL, NYMC
	MurIgc-up2	TGCTGACTCAAAATGTTAAAATGATTG	MurIgc-dn2	GATGATAATTCACCGTTAATGTCAAAATAG		QIMR
mutS	MutSgc-up	GAAGAGTCATCTAGTTTAGAATACGAT	MutSgc-dn	AGAGAGTTGTCACTTGCGCGTTTGATTGCT	2031544-2031140	UL, NYMC, QIMR
recP	RecPgc-up	GCAAATTCTGGACACCCAGG	RecPgc-dn	CTTTCACAAGGATATGTTGCC	1668713-1668255	UL, NYMC, QIMR
xpt	Xptgc-up	TTACTTGAAGAACGCATCTTA	Xptgc-dn	ATGAGGTCACTTCAATGCCC	882848-883297	UL, NYMC. QIMR
atoB	AtoBgc-up	ACGTTGCTCAGAAATATGGCAT	AtoBgc-dn	AAAGTGTTGCTAGTCCTCTGGTTAC	1634373-1634806	UL, NYMC, QIMR

[a] Based on nucleotide position in the GGS_124 genome (GenBank number AP010935).

[b] UL, University of Lisbon; NYMC, New York Medical College; QIMR, Queensland Institute of Medical Research.
