# Supplementary figures and images for: Population Genetics of Streptococcus dysgalactiae Subspecies equisimilis Reveals Widely Dispersed Clones and Extensive Recombination
Source: PLoS One. 2010 Jul 23;5(7):e11741. doi: 10.1371/journal.pone.0011741 (PMC2909212; doi:10.1371/journal.pone.0011741)

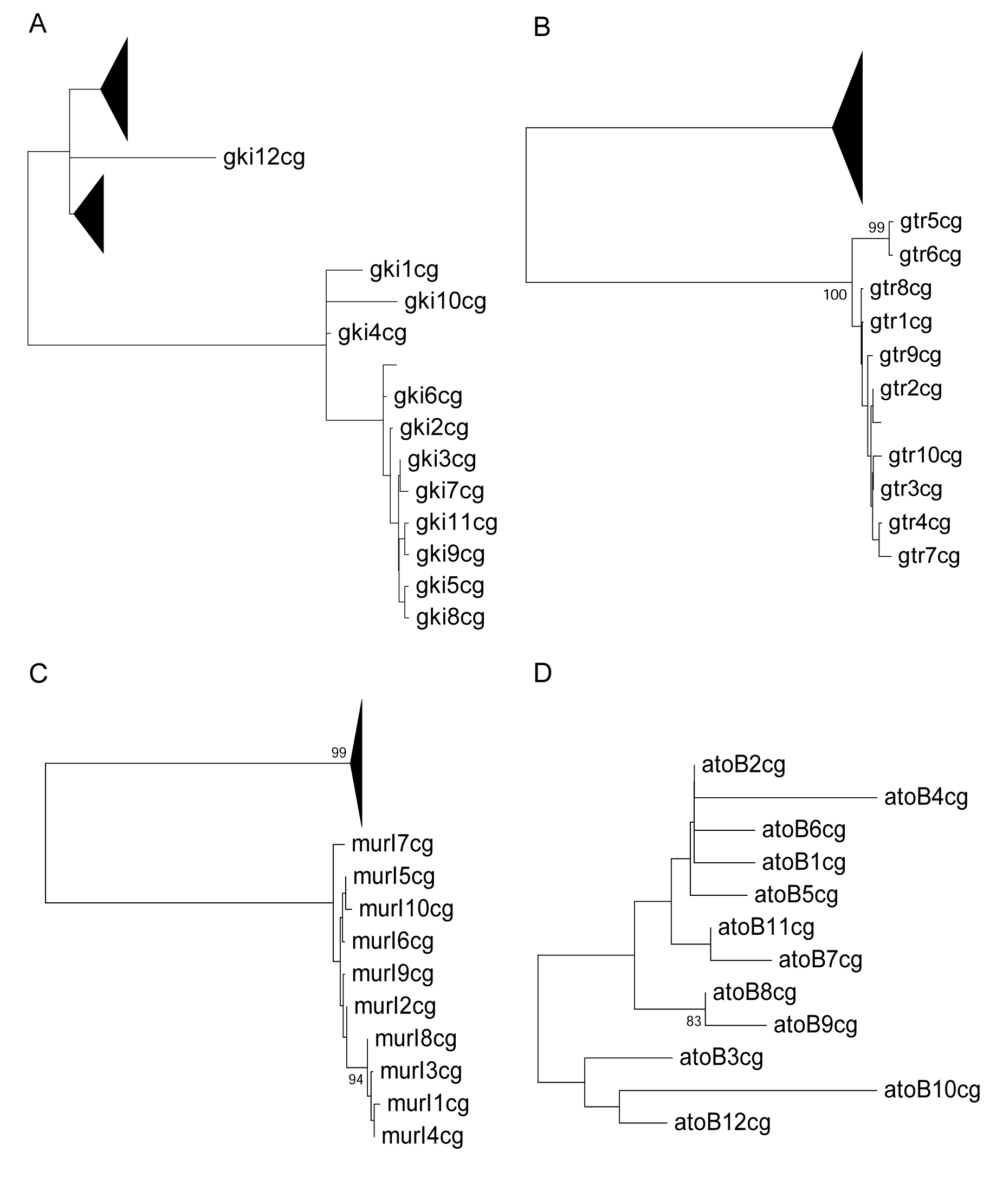

Supplement: Figure S1 — Evolutionary history of gki, gtr, murI and atoB alleles from SDSE and GAS. Evolutionary relationships were inferred using the Neighbour-Joining (NJ) method, and evolutionary distances calculated using the the Jukes-Cantor method. Branches with bootstrap support (n = 1000) greater than 80% are shown next to their respective branches. The tree is drawn to scale, with branch lengths in the same units as those of the evolutionary distances used to infer the phylogenetic tree. Only names of the GGS alleles are shown. Phylogenetic analyses were conducted in MEGA4. (0.12 MB TIF) [file pone.0011741.s003.tif]

A

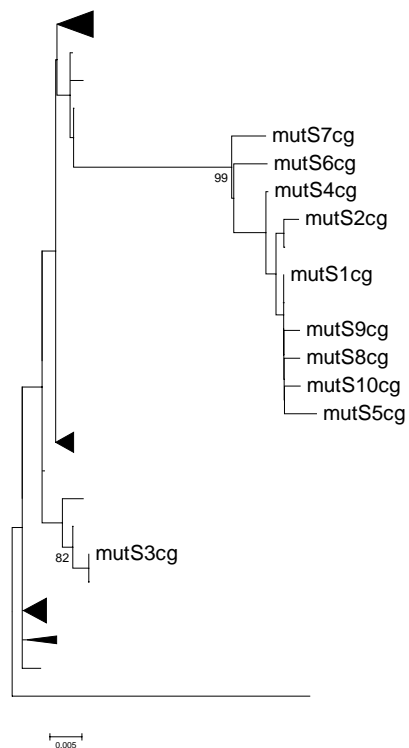

B

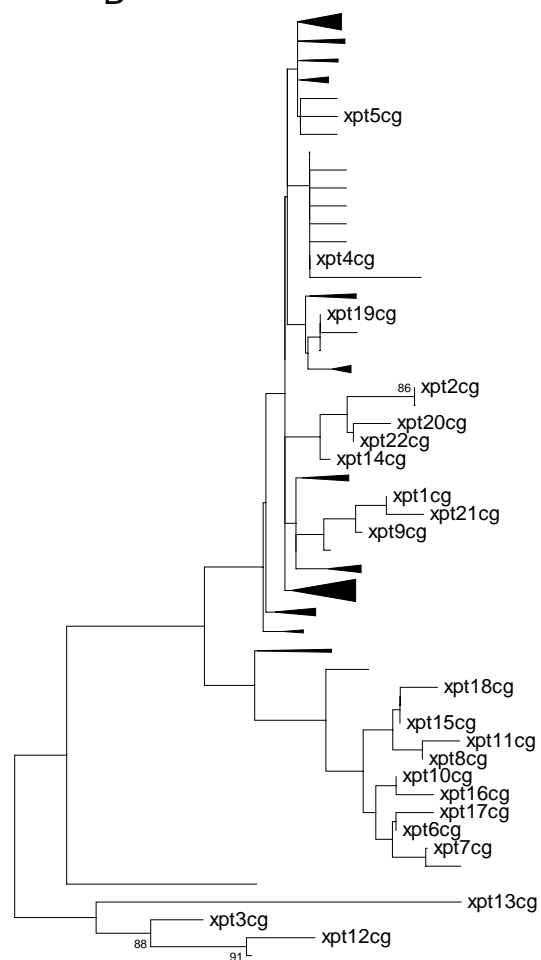

C

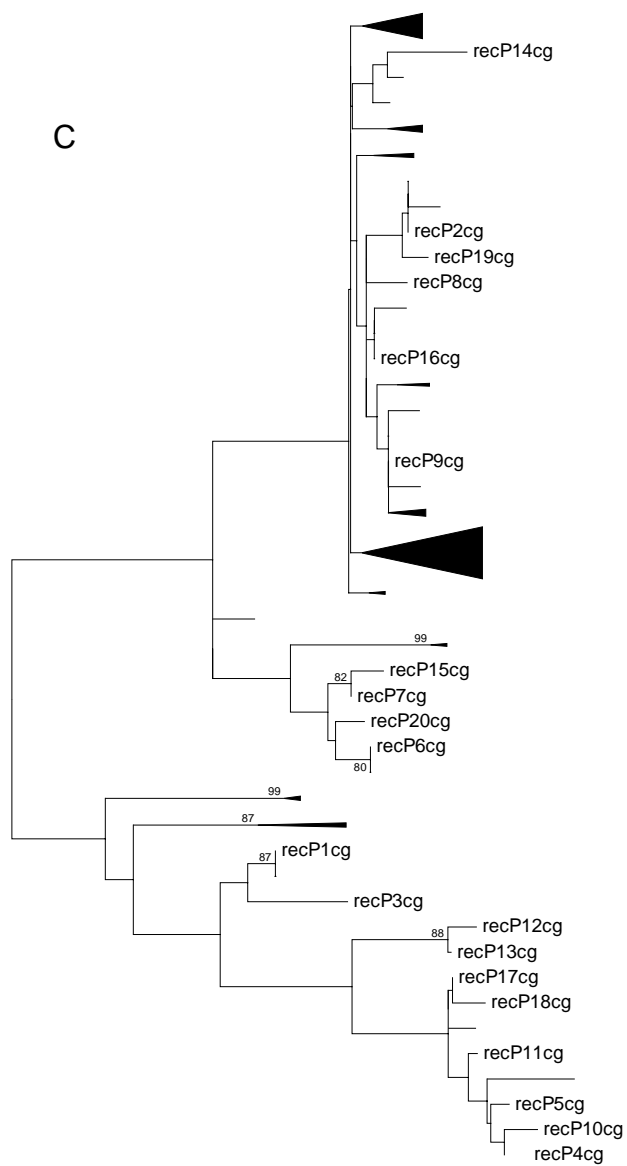

Supplement: Figure S2 — Evolutionary history of mutS, xpt and recP alleles from SDSE and GAS. Evolutionary relationships were inferred using the Neighbour-Joining (NJ) method, and evolutionary distances calculated using the the Jukes-Cantor method. Branches with bootstrap support (n = 1000) greater than 80% are shown next to their respective branches. The tree is drawn to scale, with branch lengths in the same units as those of the evolutionary distances used to infer the phylogenetic tree. Only names of the GGS alleles are shown. Phylogenetic analyses were conducted in MEGA4. (0.02 MB PDF) [file pone.0011741.s004.pdf]

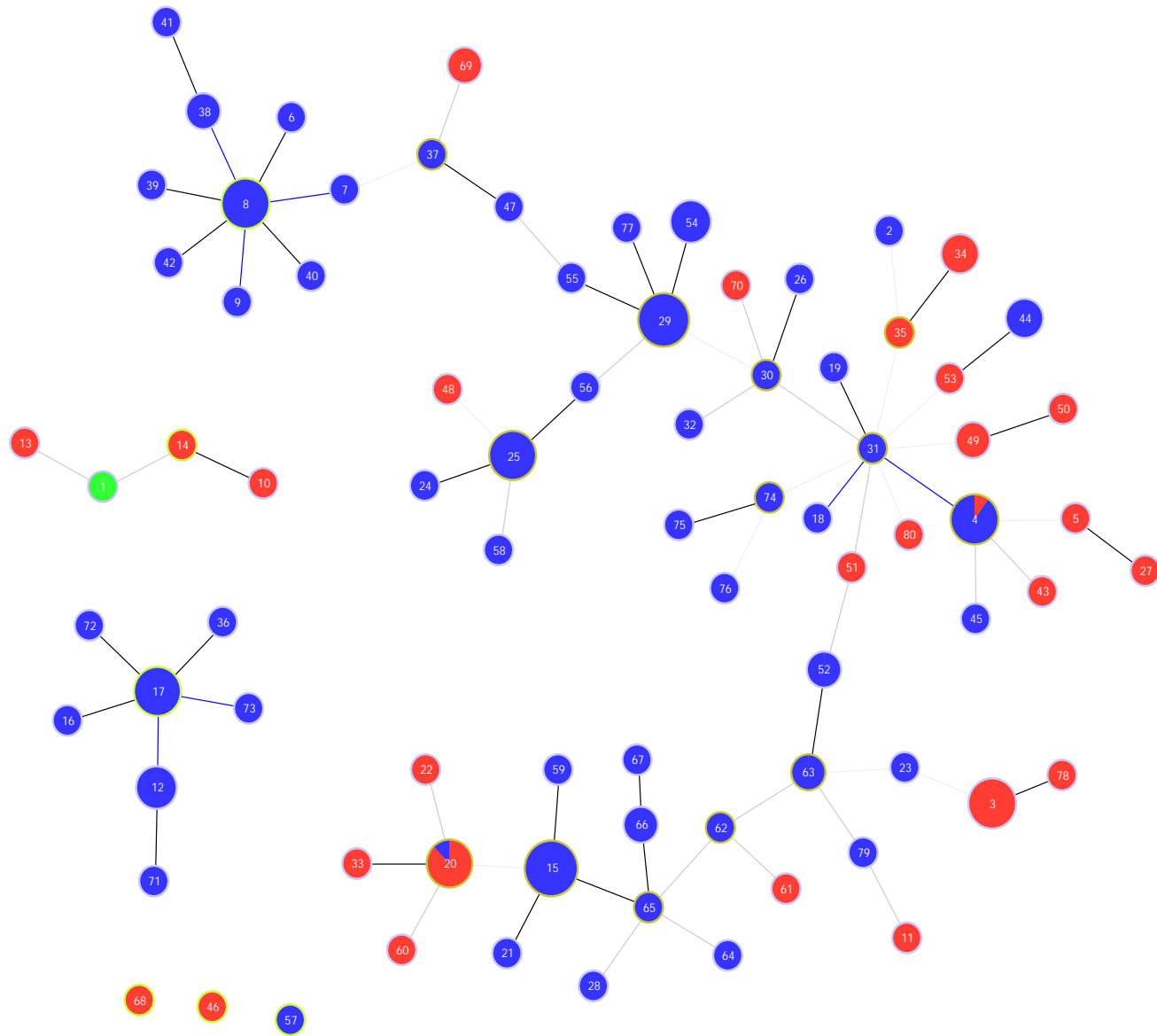

Supplement: Figure S3 — goeBURST diagram of the relationships between 178 global SDSE isolates grouped up to TLV. The size of each circle is proportional to the number of isolates with that particular ST in a logarithmic scale. STs differing up to three alleles (triple-locus variants - TLVs) are linked by straight lines. Black lines link STs differing at a single gene. Intermediate grey lines link STs differing at a two genes. Light grey lines link STs differing at a three genes. Blue circles represent isolates that have the group G carbohydrate. Red circles represent isolates expressing the group C carbohydrate. Whenever isolates of the same ST have different group carbohydrates, the number of isolates bearing the same carbohydrate is proportional to the respective color. The green circle represents the single isolate expressing the group L carbohydrate. The proposed founders of particular clusters are indicated by a light green outer circle. The sub-founders (defined has having links to three or more STs) are indicated by dark green outer circles. (0.02 MB PDF) [file pone.0011741.s005.pdf]

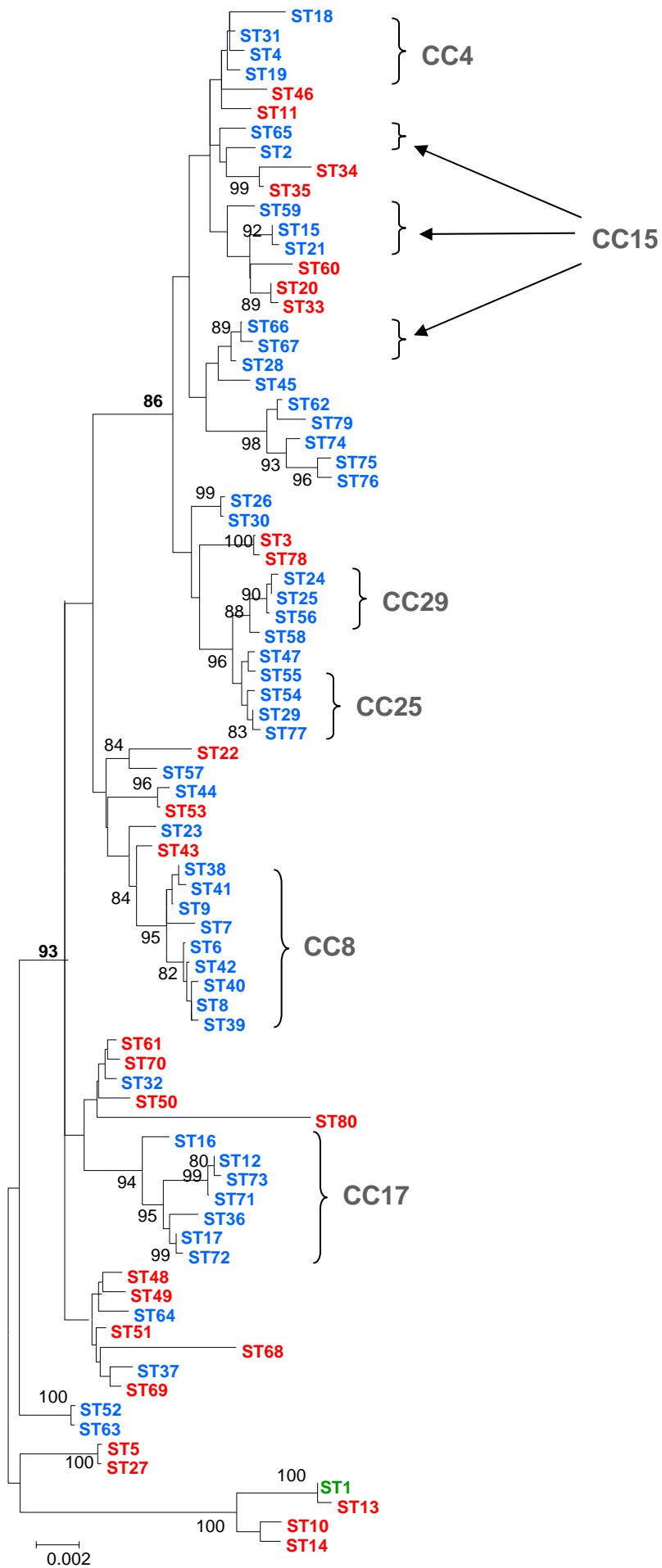

Supplement: Figure S4 — Neighbor joining tree of concatenated housekeeping alleles. The housekeeping alleles for each of the 80 STs for SDSE were concatenated (3,134 nt positions), and a neighbor joining tree was constructed (MEGA4). Bootstrap values (1,000 replicates) showing branch support equal or greater than 80% are indicated STs representing GCS and GGS are depicted in red and blue, respectively; the single group L isolate (ST1) is depicted in green. CCs having three or more STs are indicated. (0.06 MB PDF) [file pone.0011741.s006.pdf]
